# Trends in US Live Births by Race and Ethnicity, 2016-2024

**DOI:** 10.1001/jamanetworkopen.2025.56659

**Published:** 2026-01-30

**Authors:** Amos Grünebaum, Frank A. Chervenak

**Affiliations:** 1 Northwell Health, New Hyde Park, New York; 2 Zucker School of Medicine at Hofstra/Northwell, New Hyde Park, New York

## Abstract

This cross-sectional study analyzes US birth data from 2016 to 2024 to quantify changes by race and ethnicity and their implications for future maternal health care planning.

## Introduction

US demographic shifts, including immigration and declining fertility, are transforming the childbearing population. However, current policy and financing often do not keep pace with these trends. This study analyzes national birth data from 2016 to 2024 to quantify these changes and their implications for future maternal health planning.

## Methods

This cross-sectional study used the Centers for Disease Control and Prevention Wide-Ranging Online Data for Epidemiologic Research (WONDER) Natality database to analyze all live births in the US from January 1, 2016, through December 31, 2024, using data from the National Vital Statistics System.^1,2^ The dataset included 33 188 523 births. Maternal race and Hispanic ethnicity were self-reported and categorized into 7 mutually exclusive groups. We calculated the percentage of total births for each racial and ethnic group annually. To assess temporal trends, we used linear regression to determine the mean annual percentage point change (slope) and the coefficient of determination *R*^2^ to assess trend consistency. Statistical significance was defined as a 2-sided *P* < .05. Data analysis was performed using Excel, version 16.0 (Microsoft Corp) and Python, version 3.9 (Python Software Foundation). Institutional review board approval was not required because the data are deidentified and publicly available, and the study followed the STROBE reporting guideline.

## Results

From 2016 to 2024, the total number of annual live births in the US decreased by 8.4%, from 3.9 million to 3.6 million. (Figure) This period was defined by a significant demographic crossover. Births to non-Hispanic White individuals decreased from 52.6% to 49.6% of the total, decreasing below 50% for the first time (Table). This group exhibited a consistent negative linear trend (slope = −0.3 percentage points [pp]/y; *R*^2^ = 0.84).

**Figure.**
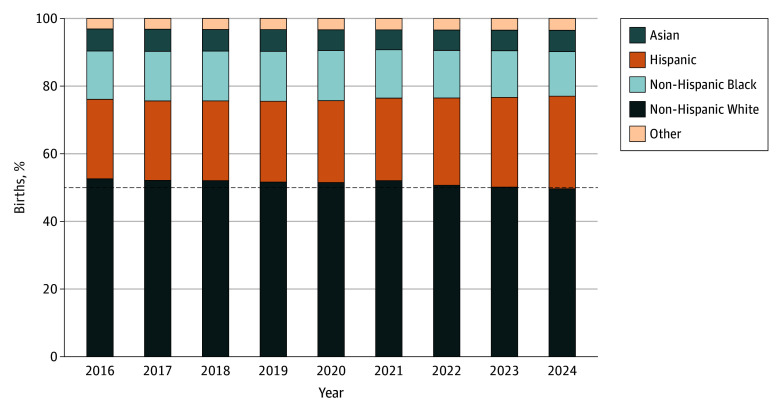
Trends in Distribution of US Live Births by Maternal Race and Ethnicity, 2016-2024 Data are from the National Vital Statistics System. Stacked bars illustrate the annual percentage of total US live births for each racial and ethnic group. The dashed horizontal line marks the 50% threshold; the proportion of births to non-Hispanic White individuals decreased below this level (49.6%) in 2024. The other category comprises American Indian or Alaska Native, Native Hawaiian or Other Pacific Islander, and multiracial births.

**Table.** US Live Births by Maternal Race and Hispanic Ethnicity, 2016 vs 2024^a^

Group	No. (%)		Absolute change, percentage points per year	Slope, percentage points per year	*R* ^2^
	2016 (N = 3 909 556)	2024 (N = 3 583 040)			
American Indian or Alaska Native	31 451 (0.8)	23 802 (0.7)	−0.1	−0.02	0.98
Hispanic (any race)	918 426 (23.5)	981 244 (27.4)	3.9	0.5	0.87
Native Hawaiian or Other Pacific Islander	9337 (0.2)	10 071 (0.3)	0.1	0.01	0.92
Non-Hispanic Asian	254 471 (6.5)	226 538 (6.3)	−0.2	−0.1	0.45
Non-Hispanic Black	558 622 (14.3)	471 928 (13.2)	−1.1	−0.2	0.55
Non-Hispanic White	2 056 332 (52.6)	1 778 191 (49.6)	−3.0	−0.3	0.84
>1 Race	80 917 (2.1)	91 266 (2.5)	0.5	0.1	0.99

^a^ Data are from the National Vital Statistics System. Slope represents the mean annual percentage point change determined by linear regression (2016-2024). *R*^2^ indicates the coefficient of determination for the linear trend.

Conversely, births to Hispanic individuals (any race) increased from 23.5% to 27.4%, the only major cohort to increase in both absolute number and proportional share (Table). This increase followed a robust positive trajectory (slope = 0.5 pp/y; *R*^2^ = 0.87). Trends for other racial and ethnic groups largely trended downward; non-Hispanic Black births decreased from 14.3% to 13.2% (slope = −0.2 pp/y; *R*^2^ = 0.55), and non-Hispanic Asian births decreased slightly from 6.5% to 6.3% (slope = −0.1 pp/y; *R*^2^ = 0.45). Among smaller cohorts, births to individuals identifying as more than 1 race demonstrated a highly consistent increase (slope = 0.1 pp/y; *R*^2^ = 0.99), while American Indian or Alaska Native births decreased (slope = −0.02 pp/y; *R*^2^ = 0.98) and Native Hawaiian or Other Pacific Islander births remained stable (slope = 0.01 pp/y; *R*^2^ = 0.92).

## Discussion

This analysis documents a major demographic transition; non-Hispanic White births now constitute less than half of US births, while Hispanic births exceed one-fourth. These shifts reflect declining fertility across most groups, contrasted with immigration trends and younger ages among Hispanic women that sustain overall birth rates.

This diversification occurs amid persistent health care workforce shortages and maternity unit closures in underserved regions. Hispanic and Black women, populations with the highest maternal morbidity and mortality,^3^ now account for over 40% of US births. Consequently, hospitals dependent on Medicaid should urgently strengthen bilingual and culturally competent care to prevent widening regional disparities.^4^

Medicaid finances over 40% of all births,^5^ and proposed reductions to eligibility or postpartum coverage would disproportionately harm mothers of racial and ethnic minority groups, potentially reversing improvements in maternal mortality rates.^6^ Policymakers need to recognize that demographic changes will magnify the consequences of safety-net retrenchment.

A limitation of this study is that broad racial and ethnic aggregations may mask subgroup heterogeneity. Strengths include the large sample size. Although limited by the absence of individual-level socioeconomic data, these robust national trends underscore an imperative. Sustained Medicaid coverage and enhanced surveillance are important to align maternal health policy with the reality of the nation’s evolving population structure.

## Notes

Supplement.
Data Sharing Statement
