# Supplementary material for: Trends in US Live Births by Race and Ethnicity, 2016-2024
Source: JAMA Netw Open. 2026 Jan 30;9(1):e2556659. doi: 10.1001/jamanetworkopen.2025.56659 (PMC12859716; doi:10.1001/jamanetworkopen.2025.56659)
Supplement: Supplement. — Data Sharing Statement [file jamanetwopen-e2556659-s001.pdf]

Data Sharing Statement

Grünebaum. Trends in US Live Births by Race and Ethnicity, 2016-2024. *JAMA Netw Open*. Published January 30, 2026. doi:10.1001/jamanetworkopen.2025.56659

Data
Data available: Yes
Data types: Deidentified participant data
How to access data: CDC WONDER database
When available: With publication

Supporting Documents
Document types: None

Additional Information
Who can access the data: All
Types of analyses: Any purpose
Mechanisms of data availability: Online
